# Children of Parents with a Mental Illness: Predictors of Health-Related Quality of Life and Determinants of Child–Parent Agreement

**DOI:** 10.3390/ijerph18020379

**Published:** 2021-01-06

**Authors:** Alina Radicke, Claus Barkmann, Bonnie Adema, Anne Daubmann, Karl Wegscheider, Silke Wiegand-Grefe

**Affiliations:** 1 Department of Child and Adolescent Psychiatry, Psychotherapy and Psychosomatics, University Medical Center Hamburg-Eppendorf, 20251 Hamburg, Germany; barkmann@uke.de (C.B.); b.filter@uke.de (B.A.); swiegand-grefe@uke.de (S.W.-G.); 2 Department of Medical Biometry and Epidemiology, University Medical Center Hamburg-Eppendorf, 20251 Hamburg, Germany; a.daubmann@uke.de (A.D.); k.wegscheider@uke.de (K.W.)

**Keywords:** children’s health-related quality of life, parents, mental disorder, child–parent agreement, children of parents with a mental illness, family psychology

## Abstract

(1) Background: Health-related quality of life (HRQoL) is frequently reduced in children of parents with a mental illness (COPMI). Child self- and parent proxy-ratings vary with raters’ characteristics and facets of HRQoL. This study aimed at analyzing risk and protective factors associated with HRQoL in COPMI, and at examining the magnitude, direction, and predictors of child–parent agreement. (2) Methods: Analyses were based on baseline data of the German CHIMPS (children of parents with a mental illness) project with *n* = 134 parents diagnosed with mental illness and *n* = 198 children and adolescents aged 8 to 18 years. (3) Results: Both children and parents reported significantly lower HRQoL than the reference population, particularly for the child’s physical and psychological well-being. Parents’ proxy-report indicated a lower HRQoL than the children’s self-report. Child and parental psychopathology, social support, and the child’s age significantly predicted HRQoL. Interrater agreement was satisfactory and better for observable aspects like physical well-being and school environment. The child’s gender-identity and mental health significantly predicted child–parent agreement. (4) Conclusions: Parental psychopathology significantly reduces children’s HRQoL. Interventions should promote resilience in children by targeting risk and protective factors. Child–parent agreement emphasizes the need to obtain both self- and proxy-reports, whenever possible.

## 1. Introduction

About one in five minor children has at least one parent with a mental illness [1]. Mental illness in primary caregivers can impair the psychosocial development of the offspring. The psychological burden of parental mental illness may not only lead to emotional and behavioral difficulties in children, but also has a more general influence on the children’s social relationships, interests, and academic environment, and thus may affect the children’s overall well-being and life satisfaction. Health-related quality of life (HRQoL) has been increasingly considered as an outcome criterion for children and adolescents to determine the burden of such demanding family conditions [2]. It has been defined as a subjective, multidimensional construct that compromises physical, psychological and social well-being [3]. Research has consistently shown that HRQoL of COPMI is reduced across different types of parental mental illness [4,5,6,7,8]. To prevent adverse psychosocial consequences for COPMI and to improve their HRQoL, it is crucial to examine risk and protective factors that are linked to the children’s well-being. Results can help to develop more efficient clinical interventions. Although self-reports are valuable sources of information, parent proxy-reports are often used as a replacement [9]. When parents suffer from mental disorders, they tend to assess their offspring’s HRQoL lower than the children do [10]. Discrepancies between children’s self- and parents’ proxy-reports can also originate from the raters’ relationship and demographic characteristics (e.g., age, gender-identity), as well as from the observability of the HRQoL domain. Investigating the extent of child–parent agreement and to identify predictors of disagreement is crucial, especially when parents are responsible to make health care decisions for their children, and when their perspective on child HRQoL differs from the child’s own rating [9,11].

### 1.1. Predictors of HRQoL in COPMI

The dynamic interaction between both risk and protective factors determines the children’s ability to adapt and recover from adverse psychosocial outcomes associated with parental mental illness [12,13,14]. Some of the most relevant risk and protective factors of HRQoL in COPMI include symptom severity of parental psychopathology and disease coping, emotional and behavioral difficulties in COPMI, the family’s mental health literacy, family functioning, social support, and child-related demographic variables. Parental psychopathology has implications for all family members. COPMI are more likely than their peers to experience unstable home environments, family conflicts, and a higher daily strain [15]. Depending on the nature and severity of symptoms, parenting skills can be impaired due to psychopathology and may result in reduced involvement with the child, insensitivity, hostility, rejection, neglect, and potential abuse [15,16]. Difficulties in parenting can also lead to insecure attachment, emotional dysregulation, negative emotionality, and pathological coping strategies, as well as psychopathology in the offspring irrespective of the children’s age [16,17]. Difficulties in parenting have been observed across different types of mental disorders, although most research has been conducted on depression. Parental depression has been associated with a markedly diminished interest in most activities, lack of energy, irritability and depressed mood, which tend to manifest in less child–parent interactions characterized by reduced empathy, verbal communication, and emotional availability, as well as a negative family discord [18,19,20,21]. The way parents appraise and cope with stressors like mental illness has both an impact on their own [22,23] and their offspring’s mental health and quality of life [24]. Research suggests that parents with a mental illness who practice adaptive coping strategies show better adaptions to their mental health condition [22], mitigate the negative outcomes of family burden and stigmatization [25], and improve HRQoL in their offspring [24].

COPMI have a significantly higher psychiatric risk than children with healthy parents due to various genetic and psychological vulnerabilities [26]. When children suffer from psychiatric symptoms, quality of life is poor and even lower compared to physical samples [27,28]. The World Health Organization (WHO) concluded based on a survey with over 51,507 participants that children with one parent with a mental illness have a 1.8 to 2.9 (odds ratio) times higher general psychiatric risk than the general population. When both parents were affected, the risk even raised from 2.2 to 4.6 (odds ratio) [29]. COPMI have a seven-fold risk to somaticize [30] and express psychiatric symptoms by physical complaints like headaches, fatigue, or stomachaches [8], which lowers satisfaction with physical aspects of HRQoL [8,29,30,31,32]. Caregivers’ depressive symptoms also reduce a child’s health-promoting behavior like healthy eating and exercise [32], which may result in dissatisfaction with physical activities and health. High health literacy in parents, which is characterized by a high amount of knowledge about the recognition, management, and prevention of mental disorders, can serve as a protective factor for the children’s mental health and promote their resilience [33].

Family functioning is an important determinant of quality of life in children and adolescents [34,35,36]. Research has consistently shown that family burden is higher in families with parental psychopathology across various psychiatric diagnoses e.g., depression [37], bipolar disorder [38], psychosis [39], and anxiety disorders [40]. Parental psychopathology may be associated with family discord, lower levels of expressiveness and affective involvement, impaired communication [37,38,39,40], and adverse psychosocial outcomes like unemployment and financial difficulties that strain family relations [15]. The extent of family burden has been determined by clinical characteristics such as symptom type and severity, a higher relapse frequency, and the severity of impaired functioning [41].

In line with the stress-buffering hypothesis, social support has been positively associated with HRQoL and psychological well-being and in children and adolescents [34,42,43]. Social support from extrafamilial sources may increase in importance, when family functioning is low. However, especially children from conflict-ridden families had difficulties to find and maintain friends and were viewed less favorably by their peers [44]. About one third of families with parental psychopathology perceive the social support they receive as insufficient [30].

The most consistent results regarding child-related demographic predictors of HRQoL exist regarding the children’s age and gender-identity. In a sample with 22,827 European participants, 8–11 years old children reported higher HRQoL than adolescents aged 12–18. Boys reported higher HRQoL than girls in most HRQoL aspects [45]. Similar age-related decreases in life satisfaction and gender-identity-related differences, especially during adolescence, have been reported in other studies [6,8,46].

### 1.2. Interrater Agreement on Child HRQoL Measures

Self-reports are generally the principle method with regard to the assessment of subjective experiences of health and well-being [34]. Nonetheless, it is still common that parents provide proxy-reports on their children’s HRQoL, whereas the children’s perspective is either neglected or surveyed only in addition [9]. This practice has been justified for younger age groups by the assumption that younger children lack sufficient cognitive and linguistic abilities to understand and interpret HRQoL questions by themselves [9]. They may also lack the ability to adopt a long-term perspective of events and consequences and have a restricted attention span [34]. Contrary to these assumptions, studies have demonstrated that even young children, who are given the opportunity to assess their own HRQoL with age-appropriate instruments, are able to understand questions and produce valid and reliable answers from the age of eight years onwards [47].

Interrater agreement on standardized child HRQoL measures may vary due to child and parent characteristics as well as with the HRQoL domain of interest. Research indicates that the child’s mental and physical health is linked to interrater-agreement on HRQoL measures [9,48,49]. Parents of healthy children over-report the children’s HRQoL compared to parents of children with physical or mental illness [9,48,49]. Parents of children with chronic conditions under-report their offspring’s quality of life [50]. When children suffer from physical rather than mental illness, child–parent agreement on HRQoL measures is higher [51], probably due to the better observability of physical symptoms [9,49,52]. Inconsistent results have been reported for child-related age and gender-identity effects on child–parent agreement [53,54,55,56]. It has been suggested that interrater agreement may vary for certain HRQoL domains like physical or emotional well-being in different developmental stages, thereby explaining the inconsistency [49].

With regard to the parent’s characteristics, research indicates that the parent’s relationship with the child as well as own perceptions of mental health and HRQoL are more predictive of child–parent agreement than the parent’s sociodemographic attributes. High family functioning characterized by high levels of intimacy and a high amount of shared time increases concordance between children and their parents [57,58]. The higher parents assessed their mental health condition [59,60] and HRQoL [11], the higher they rated their children’s well-being too, suggesting that parents project their own feelings on judgments about their children’s functioning [53]. Parents make more accurate proxy ratings when they assess objective and observable aspects of their children’s well-being (e.g., physical functioning, externalizing behavior) and have more difficulties with subjective and invisible aspects (e.g., the children’s feelings, internalizing behavior) [45]. The discrepancies reported in emotion-focused HRQoL items appear to become more discordant in adolescence compared to younger age groups [61], probably because adolescents spend more time in extrafamilial settings and prefer to discuss emotional needs with peers [49].

There are several research gaps that we aimed to overcome with this study. First, HRQoL has predominantly been investigated in adults with physical or mental disorders, or in normative samples [8,62]. Some studies have examined the HRQoL of COPMI but have either based their conclusions on bivariate correlational research, or focused solely on a few risk factors, thereby neglecting the multidimensionality of HRQoL. The inclusion of multiple predictors and regression analyses to draw conclusions on HRQoL in COPMI is still exceptional [63]. Results from multiple regression analyses may raise awareness for COPMI and allow the development and improvement of appropriate psychological interventions. Second, no study has yet, as far as we know, systematically investigated child–parent agreement regarding the children’s HRQoL when the parents were formally diagnosed with mental disorders according to the ICD-10 classification criteria. Moreover, although research has increasingly considered the children’s perspective in the last two decades, studies have mainly assessed the children’s HRQoL with parent–proxy ratings [11] and had several methodological limitations [11,50]. Small sample sizes have frequently prevented systematic analyses beyond bivariate correlational research, thereby limiting causal inference [50]. Agreement has usually been assessed with Pearson’s product–moment correlation coefficient, although it is not a measure of agreement [52,64]. A more appropriate statistic of agreement would be the intraclass correlation coefficient (ICC) [65]. In addition, predictors of agreement have rarely been investigated in multivariate analyses, which would enable researchers to glean a more realistic picture of child–parent agreement [50].

The aims of this study were, therefore first, to compare the HRQoL of COPMI with a reference population, thereby considering the children’s and the parents’ perspective. The second objective is to examine predictors of global child-related HRQoL. The third objective was the investigation of the magnitude and direction of child–parent agreement on specific and global HRQoL. Lastly, we aimed at examining variables predicting (dis)agreement with multiple child- and family-related variables. We expected that children and their parents with a mental illness reported both lower global and specific HRQoL than the reference population. Moreover, we assumed that child and parent psychopathology, low social support, a female gender-identity, older age, family dysfunction, maladaptive coping behavior were associated with lower global HRQoL in COPMI. Furthermore, we hypothesized that child–parent agreement was only of moderate size, and that disagreement on global HRQoL was predicted by child and parental psychopathology, family functioning, parental HRQoL, and the child’s age and gender-identity.

## 2. Methodology

### 2.1. Study Design

Analyses for the present study were conducted using the baseline data (gathered 2014 to 2017) of the randomized controlled CHIMPS (children of parents with a mental illness) project conducted in Germany and Switzerland. This project collected data primarily on the mental health status and HRQoL of parents with a mental illness, their partners, and children by means of standardized psychometric questionnaires to evaluate the effectiveness of the manualized family intervention “CHIMPS”. The aims of the intervention were the reduction of psychopathology in children and the enhancement of their long-term quality of life, as well as the introduction of remarkable children and adolescents to an early intervention. A detailed description of the intervention is provided in the CHIMPS manual [66]. Parents with mental disorders were recruited in multiple German and Swiss inpatient psychiatric hospitals based on the patients’ and their families’ availability and willingness to take part. Ethical approval has been provided by the Ethics Committee of the regional Medical Association (Hamburg, Germany) under the number PV4744. All participants were informed about study aims and procedures. Their participation in the study and the family intervention CHIMPS was voluntary and confidential. Written informed consent was obtained from all adult participants. Written assents of children under 18 years and the permission of their parents were received.

### 2.2. Participants

The overall sample of the project compromised 214 families with 214 parents with a mental illness, 144 partners, and 335 children. Children outside the questionnaires’ required age range (*n* = 136), and one remaining child with more than 30% missing data were excluded. The resulting final sample under analysis included *n* = 134 parents with a mental illness and *n* = 198 children and adolescents aged 8–18. Participation required parents to be diagnosed by clinicians with at least one ICD-10 psychiatric diagnosis. Participants with acute symptoms requiring inpatient treatment were excluded and referred to acute health services. Table 1 and Table 2 display the sample characteristics.

### 2.3. Measures

Health-related quality of life. The German self- and proxy-report version of the KIDSCREEN-27 and KIDSCREEN-10 was administered [34]. It covers 27 items of five domains: physical well-being (e.g., “Have you felt fit and well?”), psychological well-being, autonomy and parents, social support and peers, and school environment on a five-point response scale (1 = not at all to 5 = extremely or 1 = never to 5 = always). The KIDSCREEN-10 index contains 10 items and is derived from the 27-item version. The index provides information about global HRQoL, whereas the subscales of the KIDSCREEN-27 differentiate between specific aspects of HRQoL. The KIDSCREEN-10 provides raw values between 10 and 50, and the KIDSCREEN-27 between 27 and 135, with higher values indicating greater well-being. T-values relied on European reference data with a Mean (*M*) = 50 and a Standard Deviation (*SD*) = 10 [34]. T-scores < 40 indicate low HRQoL, scores between 40–60 indicate medium HRQoL and values > 60 high HRQoL [34]. Both KIDSCREEN versions have good discriminatory power and internal consistency (Cronbach’s α = 0.80 to 0.84), as well as good test-retest reliability (Intra Class Correlation (ICC) = 0.61 to 0.70) [34]. In the present study, the KIDSCREEN-27 demonstrated acceptable to good internal consistencies for the child-version (Cronbach’s α = 0.65 to 0.84) and the parent-version (Cronbach’s α = 0.73 to 0.91). The internal consistency of the KIDSCREEN-10 was good for both versions (Cronbach’s α = 0.79 to 0.83).

Psychopathology in parents. The Brief Symptom Inventory (BSI) [67] is a 53-item self-report questionnaire that can be answered on a five-point response scale (0 = not at all to 4 = extremely or 0 = never to 4 = always). The Global Severity Index (GSI) was used to measure current or past level of symptomatology, the number and intensity of reported symptoms, and the perceived burden. Scores can range from 0 to 4 with higher scores indicating greater psychopathology. The GSI covers nine primary symptom dimensions (somatization, obsessive-compulsive, interpersonal sensitivity, depression, anxiety, hostility, phobic anxiety, paranoid ideation, and psychoticism). The authors reported good psychometric properties, including high internal consistency of the GSI (Cronbach’s α = 0.90) [67], which could be replicated in this study (Cronbach’s α = 0.97).

Parental coping with mental illness. The Freiburg Questionnaire of Coping with Illness (FKV-LIS) [68] generates five subscales that represent the respondent’s predominant coping style based on 23 items: depressed processing style, active problem-oriented coping, distraction and self-growth, religiosity and quest for meaning, trivialization, and wishful thinking. Respondents rate on a four-point response scale ranging from 1 = not at all to 5 = very much how often they employ each coping strategy. The authors reported an internal consistency between Cronbach’s α = 0.68 to 0.77 [68]. Internal consistency in this sample ranged from Cronbach’s α = 0.49 (religiosity and quest for meaning) to 0.72 (trivialization and wishful thinking).

Parental health-related quality of life. The EQ-5D [69] is a generic self-report HRQoL measure divided into five dimensions (mobility, self-care, usual activity, discomfort, anxiety, and depression) within three severity levels. For the present study, we calculated an index value, which assigns a single value for all hypothetical health states covered by the five dimensions. An index of 1 represents the best possible state of health, while value 0 represents the opposite. The EQ-5D is a moderately valid instrument to assess HRQoL in adults with mental disorder and has reasonable discriminative ability and reliability [69]. Here, the internal consistency of the index was Cronbach’s α = 0.28.

Psychopathology in children. The Child Behavior Checklist 4–18 [70,71] is a widely used instrument to rate maladaptive emotional and behavioral problems in children aged 4–18 years on a three-point response scale from 0 = not at all to 2 = often. The parent-version has 118 items. It generates eight syndrome scales (withdrawn, somatic complaints, anxious/depressed, social problems, thought problems, attention problems, delinquent behavior, aggressive behavior) and a total score, which was used for calculations in this study. The total score can range from 0 to 111 with higher values indicating greater psychopathology in children. Psychometric validity and reliability have been established in numerous clinical and non-clinical studies [70,71]. In the present study, the total score of the CBCL-4-18 parent version demonstrated excellent internal consistency with Cronbach’s α = 0.95.

Family functioning. The General Family Questionnaire (Allgemeiner Familienbogen; FB-A) has 40 items [72]. This study focused on the total score, which reflects overall family functioning and is the sum of seven subscales (task fulfillment, role behavior, communication, emotionality, affectivity of relations, control, values, and norms). Items are rated on a four-point response scale ranging from 0 = completely true to 3 = not true at all. The total score can range from 0 to 120. Higher scores reflect greater family dysfunction. The authors reported an internal consistency of α = 0.46–0.80, with α > 0.60 for most subscales [72], which could be replicated in this study (Cronbach’s α = 0.63 to 0.74). Here, the total score had an excellent internal consistency of α = 0.93.

Social support. The Oslo Social Support Scale (OSSS-3) [73] consists of three items asking parents to proxy-report for their children the number of close confidants, the sense of concern from other people, and the relationship with neighbors and the accessibility of practical help. The scale of the first item has been adapted for study purposes from a 4 to 5-point response scale (1 = none, 2 = 1–2, 3 = 3–4, 4 = 5–6 and 5 = more than 6). The total score is calculated by summarizing those three items. It ranges from 3 to 15 with higher scores indicating greater social support. The OSSQ-3 has demonstrated good validity and reliability in a representative sample with 2524 German participants [74]. The modified version in this study demonstrated an adequate internal consistency of α = 0.69.

### 2.4. Data Analysis

We first examined whether our sample’s HRQoL differentiated significantly from the reference population [34]. Normative data were considered for both the child- and proxy reports. As children within families were more correlated than children from different families (ICC ≥ 0.10), differences were analyzed with linear mixed models. For the same reason, the impact of multiple predictors on child proxy-rated HRQoL was evaluated with linear mixed models. Coefficients, standard errors, and *p*-values were calculated for each predictor, and overall model fit was reported. To estimate child–parent agreement on HRQoL measures, ICC estimates were calculated with a two-way mixed effects model based on single ratings. ICC estimates were defined by both consistency and absolute agreement. According to Cicchetti’s guidelines, ICC < 0.4 are classified as poor, 0.40–0.59 as fair, 0.60–0.74 as good, and 0.75–1.00 as excellent reliability [75]. Multiple linear regression was performed to analyze the impact of various predictors on total disagreement of HRQoL between children and their parents with a mental illness. Total disagreement was calculated by subtracting the children’s index from the parents’ index. Family clusters were not considered as ICC values were <0.10. The scores of both regression analyses were based on raw values assessed by parents with a mental illness. All metric predictors were grand mean-centered. Questionnaire-related missings were imputed according to the Expectation–Maximization algorithm (EM) [76]. Statistical significance was set at α ≤ 0.05 two-sided. All statistical analyses were performed using IBM SPSS 25 (IBM, Armonk, NY, USA).

## 3. Results

### 3.1. Descriptive Statistics

The sample compromised 134 families (*n* = 102 mothers with a mental illness, *n* = 32 fathers with a mental illness, *n* = 198 children and adolescents). Characteristics of parents and children are described in Table 1 and Table 2, respectively. Parents with a mental illness had a mean age of M = 41.59 (*SD* = 6.77). About half of the sample was married. Most parents had 10 to 13 years of school education. The most prominent primary psychiatric ICD-10 diagnoses were mood (affective) disorders (F30–F39, e.g., major depressive disorder), followed by disorders of adult personality and behavior (F60–F69, e.g., paranoid personality disorder), and neurotic, stress-related and somatoform disorders (F40–F48, e.g., phobias, obsessive-compulsive disorder). Less prevalent were schizophrenia, schizotypal, and delusional disorders (F20–F29, e.g., delusional disorder), mental and behavioral disorders due to psychoactive substance use (F10–F19, e.g., alcohol dependence) and behavioral and emotional disorders with onset usually occurring in childhood and adolescence (F90–F98, e.g., attention deficit hyperactivity disorder). Almost half of the parents had comorbid psychiatric ICD-10 diagnoses. Most parents reported a psychologically remarkable symptom burden (BSI raw score ≥ 0.62). Parents with a mental illness frequently employed active problem-oriented coping, followed by a depressed processing style. Parents reported moderate satisfaction with current health. The *n* = 198 participating children and adolescents had a mean age of M = 12.19 (*SD* = 3.09) years. Boys and girls were equally represented. Most of the children shared the household with their parents with a mental illness and had either biological or step siblings. The sample’s mean raw value of the CBCL 4-18 was M = 37.90 (*SD* = 25.72), and M = 10.25 (*SD* = 2.40) on the OSSS-3. Children and adolescents self-reported slightly higher HRQoL on the KIDSCREEN-10 than their parents.

### 3.2. HRQoL of Children and Adolescents from the Children’s and the Parents’ Perspective

Table 3 and Table 4 show the average values of HRQoL in COPMI from the parents’ and the children’s report, respectively. Parents’ proxy reports for their children’s global and specific HRQoL were lower than the assessments of parents from the reference sample (*M* = 50, *SD* = 10). Differences were significant for the KIDSCREEN-10 index and all KIDSCREEN-27 subscales, except for autonomy and parents (all *p* < 0.01). Mean values were rated lowest for psychological and physical well-being and highest for autonomy and parents. Parents assessed their offspring’s HRQoL lower than the children did on all aspects, except for physical well-being. On average, children self-reported both lower global and specific HRQoL than the reference population. Significant differences between the sample and the reference population were found for the index as well as for the subscales physical and psychological well-being (all *p* < 0.01). Both the children’s and the parents’ perspective can be considered to represent a medium HRQoL rating [34]. Children within families were more correlated than children from different families (ICC ≥ 0.10).

### 3.3. Prediction of HRQoL in Children and Adolescents

Table 5 shows the prediction of HRQoL in children and adolescents aged 8–18 years. Family clusters were considered in the analysis as children within families were more similar to each other than children from different families (ICC = 0.45). All calculations were based on raw values. Adding the predictors to the null model significantly improved the model fit ($\chi^{2}$(df) = 98.98 (12), *p* < 0.001). Significant predictors of HRQoL were child psychopathology, social support, the child’s age, and parental psychopathology (all *p* < 0.05). Lower HRQoL was associated with child and parental psychopathology, as well as older age in children. Social support was associated with higher HRQoL. Whereas the added child-related predictors in model 1 explained 53.41% of the residual variance, family-related predictors explained only 6.23%. The model had a significant amount of variation left unexplained by the information included. Nonetheless, the fit of model 1 was significantly better than the fit of the null model.

### 3.4. Child–Parent Agreement on HRQoL in Children and Adolescents

Table 6 displays the child–parent agreement on the children’s HRQoL. Interrater agreement was significant for all global and specific aspects of HRQoL (all *p* < 0.001). ICC consistency and absolute agreement values ranged from 0.34 (social support and peers) to 0.49 (school environment), which indicates fair congruence between ratings.

### 3.5. Predictors of Child–Parent Agrement on HRQoL in Children and Adolescents

The difference between child- and parent-reports was calculated to analyze the impact of various predictors on child–parent agreement on HRQoL in children. Predictors of total child–parent disagreement on HRQoL in children and adolescents are displayed in Table 7. Parents rated their offspring’s HRQoL lower than the children did. The multiple regression model with all seven predictors explained 19.5% of the variance in the dependent variable. When the effects of all predictors are held constant, the rater show b = 3.84 deviation points. Significant predictors of disagreement were the child’s psychopathology and the child’s gender-identity (all *p* < 0.05). The difference between child- and parent-reports was smaller, when children were male and had psychological difficulties. Age, family functioning, parents’ mental health problems, and their HRQoL did not contribute to the model.

## 4. Discussion

We aimed at analyzing the impact of various risk and protective factors on HRQoL in COPMI and at examining the magnitude, direction, and prediction of child–parent (dis)agreement. As expected, both parents and children reported considerable lower global and specific HRQoL than the reference population, although ratings still indicated medium life satisfaction [34]. Parents reported lower child HRQoL than their children on most HRQoL domains. Physical and psychological well-being were the most impaired aspects of HRQoL from both perspectives. In general, these results are in line with prior research confirming that HRQoL of COPMI is underreported by parents and lower than in the general population [4,5,6,7,8,48]. More pronounced impairments in psychological and physical aspects of HRQoL are common findings in previous studies too [8,29,30,31,32]. The results show that parental mental illness impairs many facets of HRQoL in children and adolescents. To prevent adverse outcomes in those children and to improve the children’s resilience, interventions should target risk and protective factors of HRQoL in COPMI. To identify those, we analyzed the impact of various predictors of the children’s life satisfaction. Overall, results were in line with our expectations. The most influential risk and protective predictors were child and parental psychopathology, social support, and the child’s age. Of those, only social support was associated with higher HRQoL. The magnitude and the direction of the significant effects are consistent with other research [24,27,28,42,43,45,77,78,79]. The child’s gender-identity, the interaction between the child’s age and gender-identity, family functioning, and parental coping did not contribute significantly to the model, although the direction of the effects was in line with previous research [45]. Future research may evaluate whether these findings relate to overlap between predictors, the measurement instruments, sample characteristics, or whether their influence on HRQoL is lower as indicated by previous research [24,34,35,36,45]. The results imply that clinical interventions for COPMI should primarily focus on the improvement of psychological health of both children and parents, and on the increase of the children’s supportive network (e.g., relatives, peers, professionals). Although the individual needs are diverse, research indicates that most children and adolescents prioritize learning more about their parent’s mental illness, about ways to cope with it, and confidential support that is easy to access [80].

Mental health problems may affect parents’ judgments regarding their children’s HRQoL [10]. As parents are sometimes asked to make clinical decisions for their children, it is important to investigate the extent of child–parent agreement and to find probable explanations for disagreement. Consistent with our assumptions, reliability of interrater agreement between family members was fair for most global and specific aspects of HRQoL. It was slightly lower than in a study validating the proxy-version of the KIDSCREEN-27 in the general population (ICC = 0.44–0.61) [34]. However, this is in line with studies that have linked parental stress and mental health problems to higher informant discrepancies in the assessment of psychopathology and HRQoL [28,50,59,60,81]. The highest agreement was found for HRQoL relating to the school environment and the child’s physical well-being. Family members disagreed most on the child’s social relationships with peers and friends. Parents make more accurate proxy-ratings for observable aspects of behavior or for aspects like school environment for which they can rely on external sources (e.g., teachers’ reports, grades) [45,82]. The quality of relationships with peers and the perceived social support by children are often outside the parents’ visibility, especially during adolescence [49,61]. Social support and peers were also the subscale with the lowest agreement (ICC = 0.44) in the study validating the proxy-version of the KIDSCREEN-27 in the general population [34]. The most influential predictors of child–parent (dis)agreement on HRQoL in COPMI were mental health problems in children and the children’s gender-identity. They explained 19.5% of the variance in the dependent variable. The difference between child–parent reports was lower when children were male and had emotional and behavioral difficulties. Overall, results are in line with other studies that identified similar predictors of (dis)agreement [45,49,50,57]. It has been suggested that there may be more child–parent agreement in children with mental health problems, and that the parent’s perspective can provide additional valuable information on HRQoL in these situations [54]. The child’s age, family functioning, parental psychopathology, and parental HRQoL did not contribute to the model, maybe because differences between child–parent ratings were too small to find significant effects. Although controversial results exist regarding effects of age [54,56], research has consistently demonstrated that highly functional families show higher child–parent agreement [57,58], and that the parent’s mental health [10,59,60,83] and HRQoL [11] affect the proxy-ratings of their child’s HRQoL. Differences in child–parent reports regarding the children’s HRQoL have to be anticipated and regarded as valuable information in clinical and research contexts. Parents’ perceptions may be influenced by their psychiatric symptoms, concerns, and by the burden of care-giving [52]. Moreover, children and parents may experience different situations and vary in their understanding of HRQoL, indicating the need to obtain information from multiple informants if possible [52].

This study had several limitations. Because all predictors and outcome variables were simultaneously assessed in this cross-sectional study, no temporal relationship between exposure and the outcome can be made. Longitudinal data are needed to make causal inferences. As with any regression analysis, support for the model predicting HRQoL in COPMI does not necessarily mean that the results can be generalized to other populations. The generalizability of the results to the population as a whole may further be limited due to the convenience sampling method. Results may be biased due to the reasons why volunteering participants chose to take part in the CHIMPS family intervention and others did not. Furthermore, no information was available on socioeconomic status and the children’s physical condition. The latter may be a relevant predictor for physical aspects of HRQoL. Although we measured parents’ perceived burden of symptoms, we did not differentiate between psychiatric diagnoses, symptom frequency, chronicity, prognosis, and duration of mental illness. Future studies may include these predictors to explain more variance in child HRQoL and to examine the model fit with regard to different psychopathologies.

## 5. Conclusions

Our findings suggest that HRQoL is impaired in COPMI. Interventions should concentrate on the children’s psychological and physical well-being, as these seem to be the most impaired facets. To improve these domains of children’s HRQoL, interventions should focus on the whole family. They may target the parental psychopathology with psychological interventions or provide social support for the children, as these aspects appear to be closely related to the children’s HRQoL. Offering peer support groups may be one of many options to promote resilience and wellbeing in affected children by fostering psychoeducation, coping skills, and mutual support. Physical HRQoL in COPMI may be improved by cognitive interventions like mindfulness training and relaxation techniques. The disagreement found between children and parents on some aspects of the children’s HRQoL emphasizes the need to obtain both self- and proxy-reports, whenever possible. Children should be provided the opportunity to describe their subjective view, especially when it comes to HRQoL aspects that are less observable from the outside, such as psychological well-being and social relationships with peers. Parent proxy-reports may be particularly useful when children are unable to self-report due to severe cognitive deficits or a young age, as well as when children tend to overreport high HRQoL to protect their parents. Research should continue to explore the direction and magnitude of child–parent agreement on HRQoL measures, and investigate reasons for disagreement. Results may aid clinicians to decide which HRQoL instrument is appropriate for a given sample, and in which contexts children’s self and parents’ proxy reports show high deviations.

## Figures and Tables

**Table 1:** Characteristics of the parents with a mental illness.

Characteristics	*n* (%)	M (*SD*)
**Sociodemographic data**		
Age (in years) ^1^		41.59 (6.77)
Female ^1^	102 (76.1%)	
Number of children ^1^		2.14 (0.94)
Married ^1^	75 (56.4%)	
School education ^1^		
11–13 years education	39 (30.5%)	
10 years education	60 (46.9%)	
9 years education	26 (20.3%)	
No secondary education	3 (2.3%)	
**Risk and protective factors**		
Psychiatric diagnosis (ICD-10) ^2, a^		
F10–F19	2 (1.5%)	
F20–F29	4 (3.0%)	
F30–F39	75 (56.0%)	
F40–F48	15 (11.2%)	
F60–F69	37 (27.6%)	
F90–F98	1 (0.7%)	
Comorbidity (ICD-10) ^2, a^	56 (41.8%)	
Symptom burden (BSI, raw score) ^1, b^	109 (83.2%)	1.36 (0.72)
Parental Coping (FKV-LIS, raw score) ^1, c^		
Depressed processing style		15.43 (3.79)
Active problem-oriented coping		16.12 (3.78)
Distraction and self-growth		13.85 (3.31)
Religiosity and quest for meaning		13.11 (3.38)
Trivialization and wishful thinking		7.87 (3.13)
Parental health-related quality of life (EQ-5D, raw score) ^1, d^		0.57 (0.22)

Note. *n* = 134. Questionnaire-related scores were based on raw data; for measures, see text (Measures). ^1^ based on parent self-reports. ^2^ based on proxy-ratings by clinicians. ^a^ International Classification of Diseases (ICD-10) codes: mental and behavioral disorders due to psychoactive substance use (F10–F19), schizophrenia, schizotypal, and delusional disorders (F20–F29), mood (affective) disorders (F30–F39), neurotic, stress-related and somatoform disorders (F40–F48), disorders of adult personality and behavior (F60–F69), behavioral and emotional disorders with onset usually occurring in childhood and adolescence (F90–F98); ^b^ Brief Symptom Inventory (BSI); ^c^ Freiburg Questionnaire of Coping with Illness (FKV-LIS); ^d^ parental HRQoL (EQ-5D).

**Table 2:** Characteristics of the children and adolescents aged 8–18 years.

Characteristics	*n* (%)	M (*SD*)
**Sociodemographic data**		
Age (in years) ^2^		12.19 (3.09)
Female ^2^	110 (56.1%)	
Shared household with parent with a mental illness ^2^	165 (86.4%)	
(Step) siblings ^1^	164 (82.4%)	
**Risk and protective factors**		
Mental health problems (CBCL 4-18, raw score) ^2, a^		37.90 (25.72)
Social support (OSSS-3, raw score) ^2, b^		10.25 (2.40)
Family functioning (FB-A, raw score) ^2, c^		38.63 (14.70)
**Health-related quality of life**		
Child self-report (KIDSCREEN-10, raw score) ^1, d^		37.84 (6.71)
Parent proxy-report (KIDSCREEN-10, raw score) ^2^		36.60 (5.70)

Note. *n* = 198 children. Questionnaire-related scores were based on raw data; for measures, see text (Measures). ^1^ based on child self-reports. ^2^ based on parent proxy-reports. ^a^ Child Behavior Checklist 4–18 (CBCL 4-18); ^b^ Oslo Social Support Scale (OSSS-3); ^c^ General Family Questionnaire (FB-A); ^d^ child HRQoL (KIDSCREEN-10).

**Table 3:** Average HRQoL in children from the parents’ perspective.

Categories	Model-Based		
	Adjusted Mean	95% CI	ICC
**KIDSCREEN-27 Subscale**			
Physical well-being	43.81 ***	(42.42, 45.22)	0.25
Psychological well-being	43.04 ***	(41.05, 45.04)	0.13
Autonomy and parents	48.61	(47.03, 50.20)	0.71
Social support and peers	46.63 **	(44.75, 48.51)	0.41
School environment	46.94 **	(45.18, 48.70)	0.25
**KIDSCREEN-10 Index**	44.09 ***	(42.57, 45.61)	0.40

Note. *n* = 189. CI = confidence interval, ICC = intraclass correlation coefficient; calculations were based on average T-scores and analyzed with a linear mixed model, for measures, see text (Measures). ** *p* < 0.01; *** *p* < 0.001.

**Table 4:** Average HRQoL in children from the children’s perspective.

Categories	Model-Based		
	Adjusted Mean	95% CI	ICC
**KIDSCREEN-27 Subscale**			
Physical well-being	43.23 ***	(41.69, 44.78)	0.27
Psychological well-being	46.41 **	(44.39, 48.43)	0.33
Autonomy and parents	49.57	(47.79, 51.34)	0.16
Social support and peers	48.27	(46.42, 50.12)	*n*.a. ^1^
School environment	49.13	(47.30, 50.97)	0.16
**KIDSCREEN-10 Index**	47.42 **	(45.59, 49.25)	0.33

Note. *n* = 136. CI = confidence interval, ICC = intraclass correlation coefficient; calculations were based on average T-scores and analyzed with a linear mixed model, for measures, see text (Measures). ^1^ The ICC could not be estimated and was thus set to zero. ** *p* < 0.01; *** *p* < 0.001.

**Table 5:** Prediction of HRQoL (KIDSCREEN-10, raw score) in children and adolescents aged 8–18 years.

Categories	Model 1	
	Coefficients	*SE*
**Fixed Effects**		
Intercept	30.06 ***	0.49
Child-related predictors		
Child psychopathology (CBCL 4-18, raw score)	−0.09 ***	0.01
Social support (OSSS-3, raw score)	0.72 ***	0.15
Female	0.94	0.62
Age (years)	−0.37 *	0.15
Age by gender-identity	0.10	0.19
Family-related predictors		
Parental psychopathology (BSI, raw score)	−1.62 **	0.61
Family functioning (FB-A, raw score)	−0.04	0.03
Parental coping (FKV-LIS, raw score)		
Depressed processing style	0.09	0.11
Active problem-oriented coping	−0.07	0.11
Distraction and self-growth	−0.22	0.13
Religiosity and quest for meaning	0.00	0.11
Trivialization and wishful thinking	0.16	0.13
**Random Effects**		
Variance of residuals	9.66 ***	1.98
Variance of intercepts	7.76 **	2.56
ICC	0.45	
**Model Fit**		
Deviance	1047.44	
$\chi^{2}$(df)	98.98 (12) ***	
BIC	1057.71	

Note. *n* = 183. *SE* = standard error, ICC = intraclass correlation coefficient, $\chi^{2}$(df) = chi-squared (degrees of freedom), BIC = Bayesian information criterion; all calculations were based on raw data and analyzed with a linear mixed model; all metric predictors were mean-centered; for measures, see text (Measures). * *p* < 0.05; ** *p* < 0.01; *** *p* < 0.001.

**Table 6:** Child–parent agreement on HRQoL in children and adolescents aged 8–18 years.

Categories	ICC Consistency	ICC Absolute Agreement
	ICC (95% CI)	ICC (95% CI)
**KIDSCREEN-27 Subscale**		
Physical well-being	0.46 *** (0.31, 0.59)	0.46 *** (0.31, 0.59)
Psychological well-being	0.45 *** (0.30, 0.58)	0.43 *** (0.28, 0.56)
Autonomy and parents	0.42 *** (0.26, 0.55)	0.42 *** (0.26, 0.55)
Social support and peers	0.40 *** (0.24, 0.54)	0.34 *** (0.13, 0.51)
School environment	0.49 *** (0.35, 0.61)	0.49 *** (0.35, 0.61)
**KIDSCREEN-10 Index**	0.46 *** (0.31, 0.59)	0.45 *** (0.30, 0.58)

Note. *n* = 127. CI = confidence interval, ICC = intraclass correlation coefficient; for measures, see text (Measures). *** *p* < 0.001.

**Table 7:** Predictors of child–parent disagreement on HRQoL (KIDSCREEN-10) in children and adolescents aged 8–18 years.

Fixed Effects	b	*SE*
**Intercept**	3.84 *	1.88
Child-related predictors		
Child psychopathology (CBCL 4-18, raw score)	−0.05 *	0.02
Female	−3.88 **	1.21
Age (years)	0.78	0.63
Age by gender-identity	−0.42	0.42
**Family-related predictors**		
Family functioning (FB-A, raw score)	−0.05	0.04
Parental health-related quality of life (EQ-5D, raw score)	5.37	3.16
Parental psychopathology (BSI, raw score)	0.98	1.13

Note. *n* = 124, F = 5.25. df = 7/116. Model fit: adjusted R^2^ = 19.5%. b = unstandardized coefficient *SE* = standard error, CI = confidence interval; all calculations were based on raw data and analyzed with a linear mixed model; all metric predictors were mean-centered; for measures, see text (Measures). * *p* < 0.05; ** *p* < 0.01.

## Data Availability

Data available in a publicly accessible repository.
